# Satellite cell-mediated breast muscle growth and repair: The impact of thermal stress

**DOI:** 10.3389/fphys.2023.1173988

**Published:** 2023-03-31

**Authors:** Sandra G. Velleman

**Affiliations:** Department of Animal Sciences, The Ohio State University, Wooster, OH, United States

**Keywords:** chicken, growth selection, muscle, satellite cell, temperature, turkey

Poultry exposed to prolonged periods of thermal stress exhibit negative effects on breast meat quality through altered muscle structure, increased fat deposition, and altered protein levels. Birds are homotherms and maintain their body temperature in a narrow range (Yahav, 2000; 2015). Newly hatched poults and chicks are unable to maintain a consistent internal body temperature especially when challenged with external hot or cold temperatures (Dunnington and Siegel, 1984; Modrey and Nichelmann, 1992; Shinder et al., 2007). With more temperature extremes anticipated due to climate change, it is expected that the immediate posthatch period will result in newly hatched birds being more thermally challenged. The pectoralis major (*p. major*) muscle (breast muscle) has been shown to be sensitive to temperature extremes during the immediate posthatch timeframe with permanent effects on the morphological structure of the muscle including fat content and its overall development and growth (Velleman et al., 2014; Piestun et al., 2017; Patael et al., 2019; Halevy, 2020). Furthermore, chronic heat stress in chicks has been shown to increase collagen deposition (Halevy, 2020; Patael et al., 2019) and decrease circulatory supply in the *p. major* muscle (Hadad et al., 2014; Joiner et al., 2014). Proximity to the circulatory supply is required for activity of the adult myoblast (satellite cell) population of cells (Christov et al., 2007; Rhoads et al., 2009).

Satellite cells are responsible for posthatch muscle growth, repair and regeneration of the muscle, and are associated with the quality of poultry breast meat. Satellite cells were first reported by Mauro (1961) being localized between the basal lamina and sarcolemma of a muscle fiber. Muscle fiber formation is complete prior to hatch (Smith, 1963). After hatch, further muscle fiber growth occurs through the process of hypertrophy. Hypertrophy is dependent on satellite cell proliferation, differentiation, and fusion with existing muscle fibers donating their nuclei to increase protein synthesis potential (Moss and Leblond, 1971; Cardasis and Cooper, 1975). The first week posthatch is a period of maximal mitotic activity (Mozdziak et al., 1994; Halevy et al., 2000). During this period, satellite cells are sensitive to both cold and hot temperatures in terms of their proliferation (Xu et al., 2021), differentiation (Xu et al., 2021), cellular fate (Xu et al., 2022a), and ultimately will be a primary determinant of *in vivo* muscle fiber formation which will impact meat quality. For example, the proliferation (Halevy et al., 2001; Clark et al., 2016; Harding et al., 2016; Xu et al., 2021; Xu and Velleman, 2023) and differentiation (Halevy et al., 2001; Clark et al., 2016; Harding et al., 2016; Xu et al., 2021; Xu and Velleman, 2023) of satellite cells is affected by thermal stress both cold and hot. Cold temperatures inhibit both the proliferation and differentiation of satellite cells which will result in smaller myofibers and limit muscle mass accretion especially when satellite cells have high mitotic activity during the first wk posthatch and are responsive to temperature. Clark et al. (2016) in turkeys showed a linear increase in proliferation with temperature increasing from 33°C to 43°C in a stepwise manner. Similar results in chickens were reported by Harding et al. (2016). Thermal stress especially hot temperatures can increase the conversion of satellite cells to an adipogenic fate (Xu et al., 2022a) which will be associated with altered breast meat quality through increased fat content. Thus, the cellular biology of breast muscle satellite cells is central to both the morphological structure of muscle through their regulation of muscle mass accretion and meat quality. Furthermore, the repair and regeneration of myofibers back to their original state is under the control of satellite cells. If satellite cell activation back into the cell cycle from a quiescent state is suppressed or the proliferation and/or differentiation is impaired, the repair of existing muscle fibers to their original state will be negatively impacted. Interestingly, as shown by Xu et al. (2021) in turkeys that satellite cell proliferation and differentiation has increased with selection for growth whereas the opposite has occurred in broilers (Xu and Velleman, 2023). Thus, meat-type turkeys have a greater potential to repair and regenerate myofiber damage.

Satellite cells are not a homogeneous population of cells. Schultz and Lipton (1982) were the first to report satellite cell heterogeneity with proliferation being age dependent. Satellite cell heterogeneity can take many different forms with satellite cells from different muscle fiber types expressing the genes specific to the fiber type it originated from (Feldman and Stockdale, 1991; Lagord et al., 1998). Heterogeneity of satellite cells exists in a single fiber-type muscle like the turkey and chicken *p. major* muscle that contains homogenous Type IIb fibers. McFarland et al. (1995) and Yun et al. (1997) used single cell cloning to determine that satellite cells isolated from the *p. major* muscle of one turkey exhibit different rates of proliferation and differentiation and different growth factor responsiveness.

Commercial meat type poultry, chickens and turkeys, have been selected, in part, for rapid growth and heavy weight of the breast muscle. How selection for growth has affected the growth properties of satellite cells and their responsiveness to temperature is important to the morphological structure of the *p. major* muscle and breast meat quality. Faster growing poultry in general have a higher metabolic rate and produce more heat (Chwalibog et al., 2007; Buzala et al., 2015). To further complicate thermal regulation, growth-selected poultry tend to have reduced circulatory supply in the *p. major* muscle and thus reduced dissipation of heat (Yahav, 2000; Havenstein et al., 2003; Yahav et al., 2005). In general, faster growing heavy weight meat-type broilers have reduced thermal tolerance compared to slower growing lines (Yahav, 2000).

How selection for increased muscle mass accretion of the *p. major* muscle has impacted the biological activity of the satellite cells and response to thermal stress will affect muscle growth and development including the response to myofiber damage. Xu et al. (2021) reported results from a study comparing *p. major* satellite cell proliferation, differentiation, and response to hot and cold temperature in a historical turkey line representing commercial turkeys of the late 1960s (Nestor et al., 1969) to a modern-day commercial meat-type turkey line. It was found that both hot and cold thermal stress *in vitro* affected the proliferation and differentiation of the satellite cells isolated from both the historical and the current commercial turkey *p. major* muscle. Both proliferation and differentiation were increased by heat stress and reduced with cold. The cells during proliferation were more sensitive to temperature than during differentiation with temperature during proliferation having more of an effect on myotube formation. Myotube formation is the precursor to myofiber development and thus altering the proliferation process could have long-term effects on the morphological structure of the breast muscle in turkeys. Furthermore, the growth-selected faster growing modern commercial turkey satellite cells were significantly more sensitive to hot temperatures during both proliferation and differentiation. *In vivo*, this would result in the formation of larger diameter muscle fibers, giant fibers, reducing available connective tissue spacing between muscle fibers and bundles. In an anerobic muscle like the *p. major* muscle reducing the amount of available connective tissue spacing limits the area needed for capillary supply to support satellite cell activity and remove the by-products of anaerobic respiration. As shown by Wilson et al. (1990) and Velleman et al. (2003) as muscle begins to lose spacing between muscle fiber bundles and individual myofibers, degradation of the muscle fiber structure commences. Once the fibers are damaged, the fibers must be regenerated to their original state through the activation of satellite cells. If myofiber structure is not appropriately regenerated, the fiber structure of the muscle will be replaced with connective tissue and fat through fibrosis. Since the modern commercial turkey has increased proliferation and forms larger fibers with hot temperatures, it is likely to be more prone to myofiber degenerative myopathies.

Interestingly, *p. major* muscle necrotic/fibrotic myopathies are primarily observed in heavy weight fast growing chickens and not in meat type turkeys. This raises the issue of differences between satellite cell biological activity to regenerate muscle in chickens and turkeys, and if satellite cells between chickens and turkeys have a similar response to thermal stress. In a recent study, Xu et al. (2023) compared the current commercial meat-type chicken *p. major* muscle satellite cells to cell lines isolated in the 1990s. The modern commercial chicken satellite cells had decreased proliferation and differentiation and were less responsive to both hot and cold thermal stress compared to the 1990s *p. major* muscle satellite cells. This is completely opposite to that of the modern commercial meat-type turkey which has increased proliferation and differentiation compared to older slower-growing turkeys (Xu et al., 2021). Turkey derived *p. major* muscle satellite cells from current birds are also responsive to both hot and cold thermal stress during proliferation and differentiation through both the mechanistic target of rapamycin (mTOR) (Xu et al., 2022b) and Frizzled 7 (Fzd7)-mediated wingless-type mouse mammary tumor virus integration site family/planar cell polarity (Wnt/PCP) pathway (Xu et al., 2022a). The mTOR pathway stimulates protein synthesis and enhances myofiber hypertrophy (Bodine et al., 2001; Wang and Proud, 2006), whereas the Wnt/PCP is a regulator of satellite cell migration (Fortier et al., 2008; Wang et al., 2018). The modern-day commercial meat-type chicken satellite cells are less sensitive to temperature and during proliferation are more responsive to mTOR and Fzd7 expression. Taken together, the results of Xu et al. (2023) are suggestive of the modern-day broiler satellite cells having reduced regeneration potential of damaged muscle due to decreased proliferation and differentiation and temperature sensitivity of the satellite cells.

Reduced regeneration potential of broiler *p. major* satellite cells would result in current commercial broilers being more susceptible to the negative effects of necrotic/fibrotic myopathies like Wooden breast which are not observed in turkeys. In support of the negative impact of reduced regeneration potential of broiler satellite cells, Wooden breast affected muscle is composed of a high percentage of smaller diameter myofibers with disorganized contractile sarcomeres (Clark and Velleman, 2017; Velleman et al., 2018). Based on the reduced biological activity of modern-day broiler satellite cells, the ability to regenerate damaged myofibers is reduced leading to the necrosis and subsequent fibrosis. Furthermore, it is evident that selection for accretion of breast muscle mass in both turkeys and chickens has changed the satellite cell populations from those in slower growing or historic lines with changes in proliferation and differentiation (Xu et al., 2021; Xu et al., 2023) and in the case of turkeys documented changes in key cell surface receptors affecting satellite cell function and cellular fate (Xu et al., 2023).

In summary, both thermal stress and the biological activity of satellite cells pose multidimensional threats to the growth, development, and subsequent meat quality of the poultry breast muscle. Satellite cells have their peak mitotic activity, the first week posthatch, and during this time are responsive to extrinsic stimuli including temperature which can alter cellular fate, and proliferation and differentiation. In addition, satellite cells are not a homogenous population of cells and have been altered by selection for growth. In the case of broiler chickens, satellite cell proliferation, differentiation, and the potential to regenerate myofiber damage has declined likely being associated with the onset of fibrotic/necrotic myopathies (Xu and Velleman, 2023). Also, the satellite cell response to temperature extremes has changed in both turkeys and chickens. Selection strategies used by the poultry industry as it continues to move forward should include assessments of satellite cell biological activity and responsiveness to both hot and cold temperature extremes.
